# Impact of Water–Cement Ratio on Concrete Mechanical Performance: Insights into Energy Evolution and Ultrasonic Wave Velocity

**DOI:** 10.3390/ma17153651

**Published:** 2024-07-24

**Authors:** Junzhi Lin, Bincheng Tian, Zelong Liang, Enpeng Hu, Zhaocun Liu, Kui Wang, Tao Sang

**Affiliations:** 1 Key Laboratory of Hydraulic and Waterway Engineering of the Ministry of Education, Chongqing Jiaotong University, Chongqing 400074, China; 622220960015@mails.cqjtu.cn; 2 School of River and Ocean Engineering, Chongqing Jiaotong University, Chongqing 400074, China; liuzc17@126.com (Z.L.); anhuiwk@163.com (K.W.); 3 Chongqing Municipal Key Laboratory for Disaster Reduction of Mountain Road and Water Transportation Geology, Chongqing Jiaotong University, Chongqing 400074, China; 4 Department of Hydraulic Engineering, Tongji University, Shanghai 200092, China; 5 Chongqing One Three Six Geological Team, Chongqing 401147, China; huenpeng@foxmail.com; 6 Hunan Water Planning & Design Institute Co., Ltd., Changsha 410153, China; sangtaochi@163.com

**Keywords:** uniaxial compression, crack propagation, energy evolution, ultrasonic wave velocity, damage characteristics

## Abstract

The water–cement ratio significantly affects the mechanical properties of concrete with changes in porosity serving as a key indicator of these properties, which are correlated with the ultrasonic wave velocity and energy evolution. This study conducts uniaxial compression tests on concrete with varying water–cement ratios, analyzing energy evolution and ultrasonic wave velocity variations during the pore compaction stage and comparing damage variables defined by dissipated energy and ultrasonic wave velocity. The results indicate the following findings. (1) Higher water–cement ratios lead to more complete hydration, lower initial porosity, and a less pronounced pore compaction stage, but they deteriorate mechanical properties. (2) In the pore compaction stage, damage variables defined by dissipated energy are more regular than those defined by ultrasonic wave velocity, showing a nearly linear increase with stress (*D* = 0~0.025); ultrasonic wave variables fluctuate within −0.06 to 0.04 due to diffraction caused by changes in the pore medium. (3) In the pre-peak stress stage, damage variables defined by ultrasonic wave velocity show a distinct threshold. When the stress ratio exceeds about 0.3, the damage variable curve’s growth shows clear regularity, significantly reflecting porosity changes. In conclusion, for studying porosity changes during the pore compaction stage, damage variables defined by dissipated energy are more effective.

## 1. Introduction

It is well known that the mechanical properties of concrete are influenced by various factors [1,2]. The water–cement ratio is a crucial parameter in the concrete production process, significantly affecting the mechanical properties of concrete, primarily through changes in porosity [3]. However, current studies often overlook the influence of the water–cement ratio on the pore compaction stage. There are numerous randomly distributed initial defects, such as microcracks and micropores, which are present in specimens prepared with different water–cement ratios. The irregular evolution of these initial defects determines the macroscopic mechanical properties of the specimens [4].

Nondestructive testing is extensively used in studying the mechanical properties of concrete, such as damage detection in concrete columns [5], assessment of the health status of reinforced concrete [6,7], the development and expansion of concrete cracks [8,9], and the evaluation of uniformity and stability [10], which is of great significance for ensuring the safety and durability of concrete [11]. Common nondestructive testing methods for concrete include X-ray tomography, acoustic emission tests, and ultrasonic testing. Changes in grayscale values and variances obtained through X-ray tomography can reflect the development of microcracks in concrete specimens, thereby characterizing internal damage [12]. Common nondestructive testing methods for concrete include X-ray tomography, acoustic emission tests, and ultrasonic testing. Changes in grayscale values and variances obtained through X-ray tomography can reflect the development of microcracks in concrete specimens, thereby characterizing internal damage [13]. The change in ultrasonic wave velocity obtained through ultrasonic tests can reflect the development of cracks in concrete specimens during the compression process [14]. Methods such as X-ray tomography, acoustic emission characteristics, and ultrasound can all reflect crack development and changes in concrete specimens, thereby characterizing the overall mechanical properties of concrete. However, during the pore compaction stage, the closure and development of microcracks inside the concrete specimens are not prominent. Therefore, methods such as X-ray tomography, acoustic emission characteristics, and ultrasound cannot effectively capture these changes.

Concrete frequently undergoes pressure in engineering applications. The essence of concrete material damage lies in the dynamic expansion of cracks driven by energy [15]. Therefore, the process of concrete damage is also a comprehensive procedure of energy dissipation and release [16]. The evolution of energy permeates the entire process of concrete deformation and failure. Studying the energy evolution law during the compressive failure process of concrete can reflect changes in porosity [17], which has great practical engineering implications [18,19].

Previous studies have demonstrated that the energy evolution law can more accurately reflect the mechanical properties of concrete and is beneficial for researching the brittleness, stability, fracture mechanisms, mechanical damage characteristics, and destructiveness [20,21,22]. Experimental investigations on the dynamic strength of concrete have confirmed that variations in energy dissipation amplitude can mirror the mechanical state of concrete [23]. Within the thermodynamic framework, elastic strain energy can precisely delineate anisotropy induced by internal cracks in concrete [24]. Additionally, the energy evolution law aptly reflects the development of concrete cracks [25] and alterations in its mechanical properties under diverse external environments [26,27,28]. This holds significant reference value for real-time monitoring and fault warning of concrete structures [29].

In summary, this paper conducts uniaxial compression tests on concrete with different water–cement ratios. The porosity inside the specimens was assessed through the comparison of ultrasonic signals, and the ultrasonic testing results and the energy evolution law were used for mutual verification. The energy evolution and changes in ultrasonic wave velocity during the pore compaction stage of concrete with different water–cement ratios were analyzed in detail, and the characteristics of the damage variables defined from the perspectives of dissipated energy and ultrasonic wave velocity were compared. This study aims to provide a reference for damage detection during the pore compaction stage of concrete with different water–cement ratios in practical engineering.

## 2. Experimental Materials and Methods

### 2.1. Test Material

**Figure 1:**
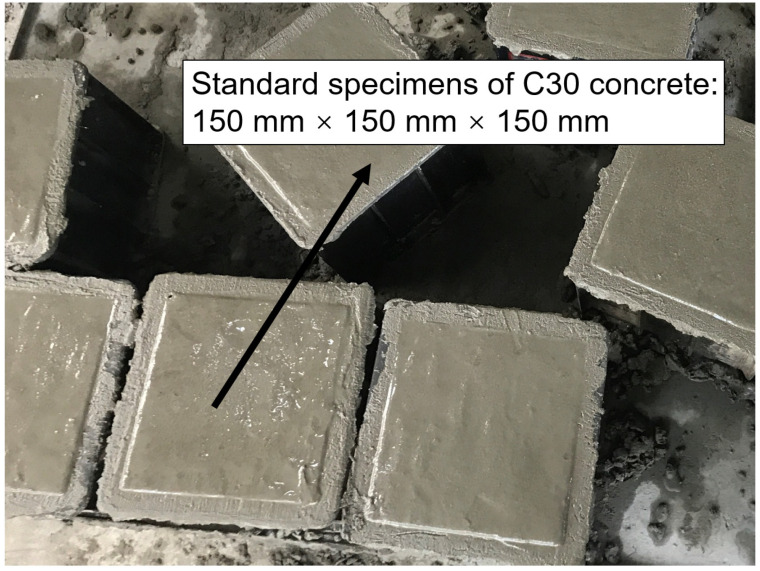
On-site diagram of the production of standard concrete specimen.

The cement used in this test was P.042.5 ordinary Portland cement from Chongqing Huaxin Fortress; its basic properties are shown in Table 1. The coarse aggregate used was limestone crushed stone with a particle size of 5 to 20 mm; its basic properties are shown in Table 2. The fine aggregate utilized was natural river sand, and tap water from the laboratory was used for mixing. According to the “Regulations for the Design of Hydraulic Concrete Mix Ratio” (DLT5330-2015) [30], C30 concrete specimens with water–cement ratios of 0.44, 0.49, and 0.54 were prepared. Each specimen measured 150 mm × 150 mm × 150 mm, with four samples fabricated for each ratio, resulting in a total of 12 cubic samples, as shown in Figure 1. The mix proportions are detailed in Table 3. Following casting, the concrete specimens underwent natural curing for 24 h before demolding. After demolding, the specimens were placed in a curing room maintained at a temperature of (20 ± 2) °C and a relative humidity exceeding 95% for 28 days.

### 2.2. Test Scheme

#### 2.2.1. Uniaxial Compression Test

**Figure 2:**
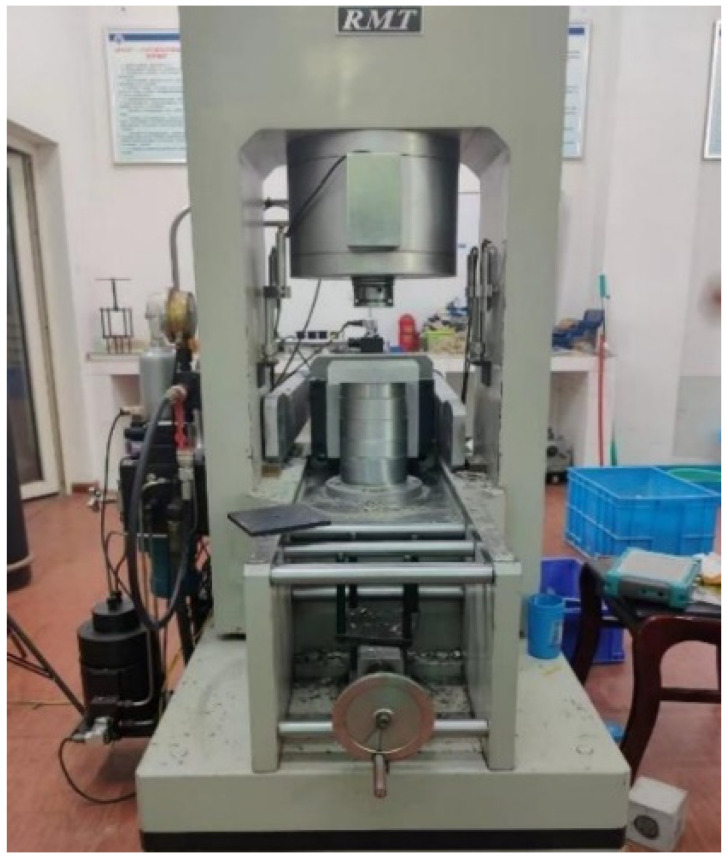
RMT-150 rock mechanics test pressure testing machine.

The test was conducted in accordance with the “Standard for Test Methods of Physical and Mechanical Properties of Concrete” (GB/T 50081-2019) [31]. The uniaxial compression test was conducted using the RMT-150 rock mechanics test machine which was designed and manufactured by Wuhan Institute of Rock and Soil Mechanics, Chinese Academy of Sciences, as shown in Figure 2. This test system enabled the acquisition of real-time stress–strain data of the specimens under compression.

After curing, uniaxial compression tests were carried out on concrete specimens with different water–cement ratios. Four sets of experiments were conducted for each water–cement ratio. The specific steps are as follows:

1. Clean the surface of the specimens and apply vaseline as a lubricant to the upper and lower ends to reduce frictional resistance and mitigate internal damage.
2. Place the test specimen on the pressure machine and adjust it so that the bearing surface aligns with the center of the upper and lower pressure plates.
3. Select the stress control loading method with an average loading rate of 1 kN/s.
4. Initiate the test instrument to apply load until the specimen fails.

#### 2.2.2. Ultrasonic Test

**Figure 3:**
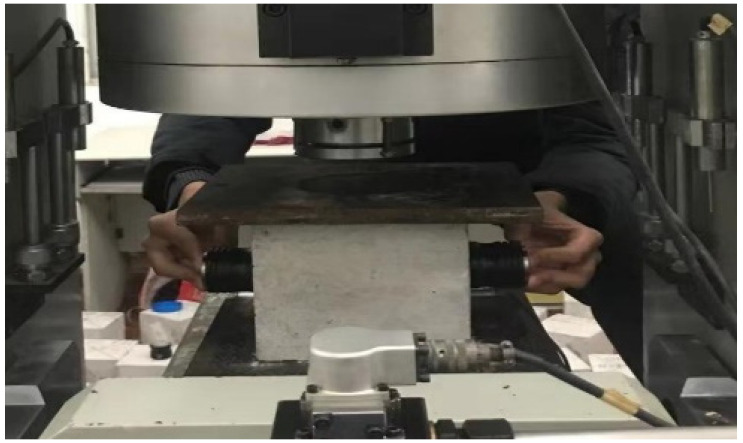
Ultrasonic field test diagram.

**Figure 4:**
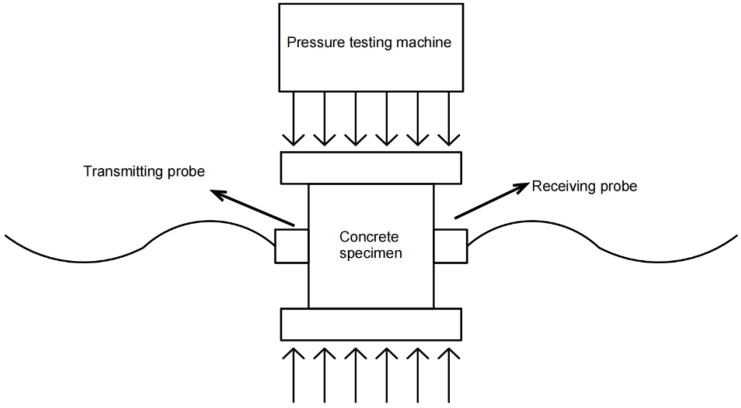
Ultrasonic test signal device layout diagram.

The RSM-SY5 (T) non-metallic acoustic detector, manufactured by Wuhan Zhongyan Science and Technology Co., Ltd. (Wuhan, China) of China, was used for the ultrasonic testing experiment, operating at an ultrasonic frequency of 50 kHz. During the uniaxial compression of the concrete specimens, ultrasonic testing was performed using the transmission method. Ultrasonic parameters were gathered every 25 kN until the specimens failed. The on-site ultrasonic testing setup is shown in Figure 3, and the schematic diagram of the arrangement of the ultrasonic signal transmitting and receiving devices is shown in Figure 4. These devices were symmetrically arranged on the sides of the compressed concrete specimen to facilitate optimal ultrasonic signal collection.

## 3. Analysis of Test Results

### 3.1. Stress–Strain Characteristic Analysis

**Figure 5:**
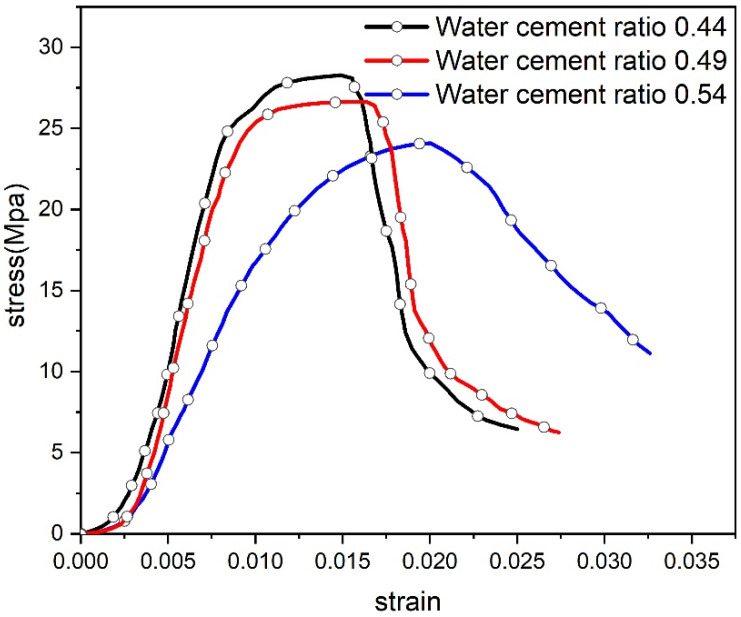
Total stress–strain curves of different water–cement ratios.

The stress–strain curve is a crucial tool for evaluating the compressive strength characteristics of concrete. Based on the results of the uniaxial compression test, the complete stress–strain curves for concrete with various water–cement ratios were plotted, as shown in Figure 5.

The water–cement ratio significantly influences the stress–strain curve of concrete. During the stress-rising stage, the slopes of the curves for concrete with different water–cement ratios are in the following order: *V*_0.44_ > *V*_0.49_ > *V*_0.54_. This indicates that the water–cement ratio affects the initial pore structure of the concrete. The peak strength and elastic modulus are highest for the concrete specimen with a water–cement ratio of 0.44 and lowest for the specimen with a water–cement ratio of 0.54. Peak stress and elastic modulus are critical parameters for gauging the deformation resistance of concrete. This finding reveals that the mechanical performance of the concrete specimen with a water–cement ratio of 0.44 is the best, followed by the specimen with a water–cement ratio of 0.49, and the poorest performance is observed in the specimen with a water–cement ratio of 0.54.

During the stress-declining stage, the stress–strain curves show that the rate of stress decline after the peak for concrete with different water–cement ratios is in the following order: *V*_0.44_ > *V*_0.49_ > *V*_0.54_. This indicates that the strength of concrete with water–cement ratios of 0.44 and 0.49 diminishes more rapidly. The increased water–cement ratio enhances the ductility of the concrete, causing the failure mode to shift from brittle to plastic.

### 3.2. Analysis of Energy Evolution Characteristics

#### 3.2.1. Law of Energy Evolution

The deformation and failure of concrete involve an exchange of internal energy exchange with external energy. According to the first law of thermodynamics, the energy of concrete specimens under uniaxial compression primarily converts into elastic energy and dissipative energy. This perspective neglects the heat exchange between the internal system and the external environment during specimen loading. The energy transformation relationship can be expressed as follows:

(1) $$U=U_{e}+U_{d}$$

In the energy transformation relationship, *U* represents the total energy of the concrete specimen, which is measured in J/cm^3^. *U*_e_ denotes the elastic energy stored within the specimen, which is also measured in J/cm^3^. *U*_d_ signifies the dissipative energy dissipated during the deformation and failure process, which is also measured in J/cm^3^.

In light of the stress–strain relationship of uniaxial compression, the correlation between elastic energy, dissipated energy, and total energy of concrete is as follows:

(2) $$U=\int \sigma d\varepsilon =\sum_{i=1}^{n}\frac{1}{2}(\sigma_{i}+\sigma_{i-1})(\varepsilon_{i}-\varepsilon_{i-1})$$

(3) $$U_{e}=\sigma^{2}/2E$$

(4) $$U_{d}=U-U_{e}$$

where *σ_i_* is the axial compressive stress of the concrete specimen at the *i*th acquisition, MPa; *ε_i_* is the axial compressive strain of the concrete specimen at the *i*th acquisition; and *E* is the elastic modulus of the concrete, Mpa.

**Figure 6:**
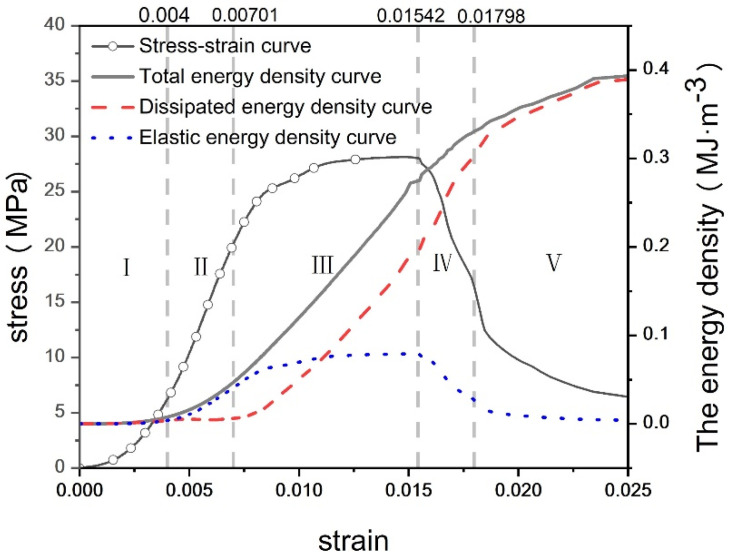
Whole-process energy evolution diagram of concrete with water–cement ratio of 0.44.

**Figure 7:**
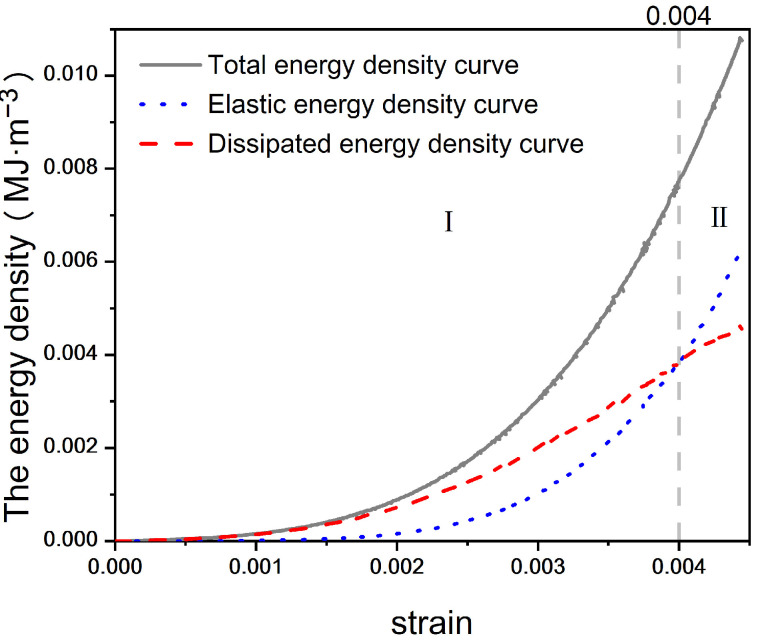
Energy evolution diagram of concrete pore compaction stage (Stage I) with water–cement ratio of 0.44.

According to the stress–strain characteristics and the law of energy evolution, the energy evolution of concrete during compression can be divided into five stages: (I) void compaction stage, (II) elastic stage, (III) plastic stage, (IV) failure stage, and (V) post-failure residual strength stage [32]. In the energy evolution characteristic curve, the elastic energy density and the dissipated energy density of concrete with different water–cement ratios exhibit the same characteristic trends as their corresponding total energy density. Therefore, concrete with a water–cement ratio of 0.44 was selected, and the corresponding energy evolution characteristic diagram was sketched based on Equations (1)–(4), as shown in Figure 6. As shown in the figure, with the increase in strain, the total energy and dissipative energy gradually increase, while the elastic energy initially rises and then declines.

(I) Pore Compaction Stage: At this stage, numerous initial pores exist within the concrete, and the energy input from the exterior is primarily used to compact these initial pores and microcracks. Most of the total external energy is transformed into dissipative energy generated by the compression of pores, making the dissipative energy greater than the elastic energy, as depicted in Figure 7.
(II) Elastic Deformation Stage: During this stage, the concrete undergoes primarily elastic deformation with the input energy predominantly stored as elastic energy. The elastic energy typically maintains a constant growth rate. Crack development inside the concrete is minimal, and the frictional heat generated by fine particles under external forces results in a small amount of dissipative energy, which increases gradually but at a slow rate.
(III) Plastic Stage: The elastic energy continues to increase albeit at a slower rate. Microcracks begin to develop, causing the concrete to behave more like a plastic material. With the appearance of cracks, the dissipative energy starts to increase significantly. However, at this point, the cracks do not fully penetrate the concrete, and it still functions as a relatively efficient energy storage material.
(IV) Failure Stage: At this stage, a significant number of microcracks develop, leading to concrete damage. The elastic strain energy stored in the concrete is released, resulting in the formation of shear cracks. Consequently, the elastic potential energy curve starts to decrease, while the dissipative energy curve increases linearly.
(V) Residual Strength Stage: After failure, as shear cracks penetrate and concrete fragments begin to spall, the specimen approaches its residual strength. The input total energy curve grows at a slower rate, eventually reaching zero growth. The dissipated energy curve gradually aligns with the total energy curve. Simultaneously, the elastic energy curve starts to decrease due to the progressive destruction of the concrete skeleton, gradually approaching zero as well.

#### 3.2.2. Energy Dissipation during the Pore Compaction Stage

**Figure 8:**
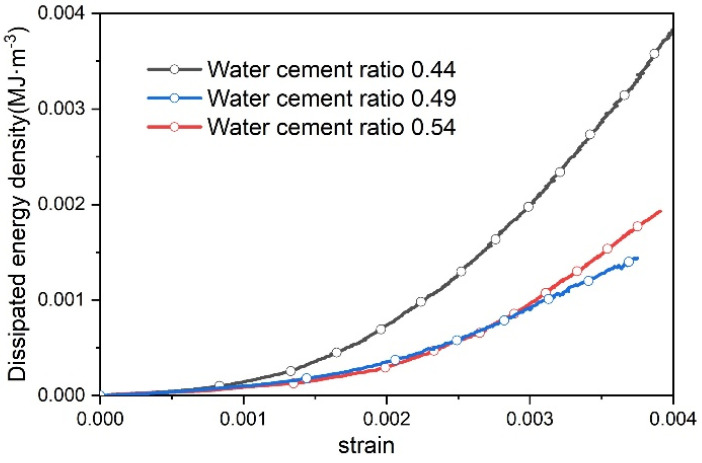
Curves of dissipated energy at pore compaction stages of different water–cement ratios.

According to the first law of thermodynamics and the correlation between elastic energy and dissipated energy, when the elastic energy conversion rate curve equals the dissipated energy conversion rate curve first, the stress–strain curve of concrete enters the linear elastic stage, marking the crack closure point [33]. Therefore, the dissipated energy density diagram during the pore compaction stage is plotted as illustrated in Figure 8.

During the compression process of concrete specimens, the development of cracks is closely related to the increase in dissipated energy [34] with internal damage resulting from energy dissipation. As shown in Figure 8, the characteristics of the pore compaction stage are more pronounced in concrete specimens with a lower water–cement ratio. In this stage, the dissipated energy curve shows a uniformly ascending trend. The concrete specimen with a water–cement ratio of 0.44 exhibits the greatest increase in dissipated energy and the most rapid growth rate, while the specimen with a water–cement ratio of 0.54 shows the smallest increase with its dissipated energy curve growth rate similar to that of the specimen with a 0.49 ratio. This is because the magnitude of dissipated energy in the pore compaction stage primarily depends on the plastic work performed by external forces in compressing the pores. Therefore, the greater the initial porosity of the concrete, the higher the dissipated energy [17].

It can be concluded that the concrete with a water–cement ratio of 0.44 has the largest porosity, followed by the concrete with a 0.49 ratio, and the concrete with a 0.54 ratio has the smallest porosity. This is consistent with the findings in the literature [33]. As the water–cement ratio increases, the fluidity of the cement paste also increases, forming a cementitious layer that blocks certain pores, thereby reducing porosity [3].

### 3.3. Analysis and Verification of Ultrasonic Wave Velocity Change

#### 3.3.1. Changes in Ultrasonic Wave Velocity

**Figure 9:**
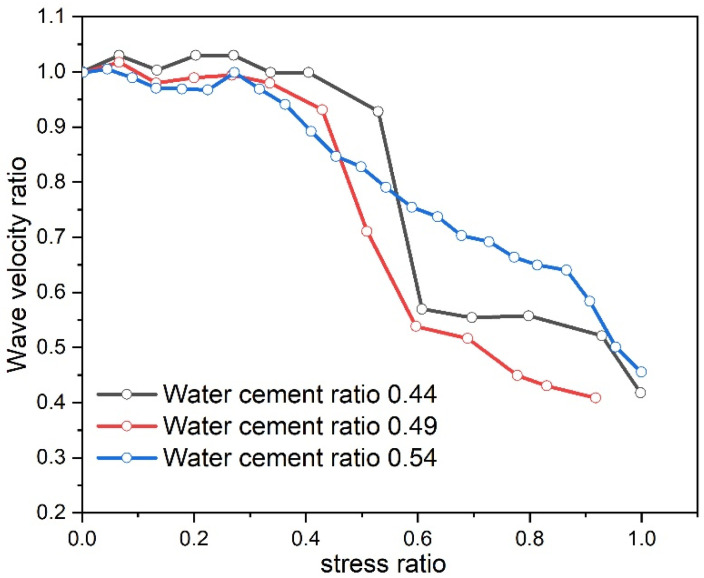
Wave velocity change in different water–cement ratio test pieces.

Ultrasonic nondestructive testing technology is frequently employed to detect flaws within concrete and monitor the progression of cracks. The alteration of ultrasonic wave velocity can infer the development of concrete cracks and assess the porosity magnitude of concrete specimens with different water–cement ratios [35]. The point measurement method is employed to measure changes in ultrasonic wave velocity during the compression process of concrete specimens. The curve of the wave velocity ratio and stress ratio of the concrete specimens under different water–cement ratio conditions is plotted, as depicted in Figure 9.

From Figure 9, it can be observed that when the concrete specimen is compressed and damaged, the overall ultrasonic wave velocity curve exhibits a downward trend, which is consistent with the reference [36]. This indicates the development of internal cracks within the concrete. The water–cement ratio significantly influences the relationship between wave velocity and stress. In the initial stage of specimen compression, the wave velocity–stress curve shows a wavelike pattern with smaller fluctuation amplitudes for specimens with a higher water–cement ratio. The ultrasonic wave velocity increases with porosity, indicating that specimens with a higher water–cement ratio have smaller porosity. As stress increases, the specimen with a water–cement ratio of 0.54 enters the descending stage the earliest and has the slowest descent rate. Conversely, the specimen with a water–cement ratio of 0.44 enters the descending stage the latest and has the fastest descent rate. This suggests that the specimen with a water–cement ratio of 0.54 begins developing microcracks earlier in the compression process but at a slower rate. In contrast, the specimen with a water–cement ratio of 0.44 develops cracks later but in larger quantities over a short period.

#### 3.3.2. Comparative Verification of Pore Compaction Stages

**Figure 10:**
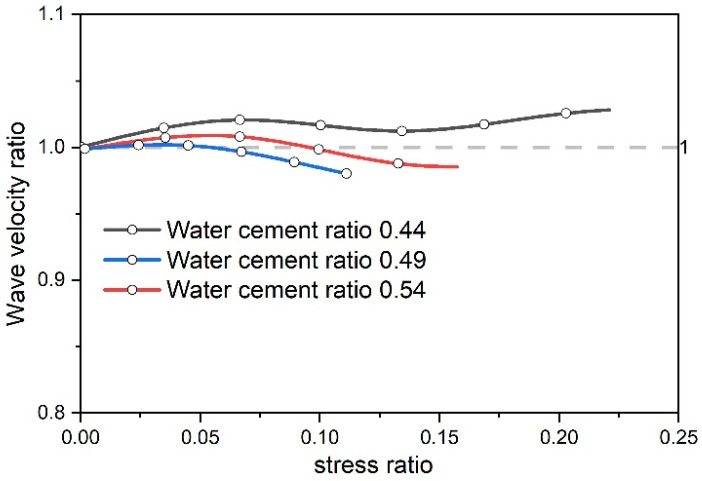
Wave velocity changes in the pore compaction stage of different water–cement ratio test pieces.

To verify the relationship between the porosity of concrete specimens with different water–cement ratios based on dissipated energy, the wave velocity ratio and stress ratio curves of concrete specimens in the pore compaction stage were plotted, as shown in Figure 10. From Figure 10, it can be observed that the wave velocity ratio curve of the concrete specimens in the compaction stage exhibits a wavelike shape. Initially, there is a minor increase in the wave velocity ratio–stress ratio curve, as the change in wave velocity is related to the medium through which the ultrasonic wave travels. As the pores in the concrete specimen are compressed, the gas within the pores decreases, and the solid medium for ultrasonic wave propagation increases, resulting in a small increment. This is consistent with the results reported in the literature [3].

As stress escalates, the concrete continues to be compressed, causing the primary fissures to repeatedly compact and close. The ultrasonic wave undergoes reflection, diffraction, and other phenomena between the fissures, leading to small changes in the wave velocity ratio [37].

Additionally, it can be discerned that the wave velocity of the concrete specimen with a water–cement ratio of 0.44 shows the most significant increase, while the specimen with a water–cement ratio of 0.54 shows the slightest increase. The alteration of wave velocity is correlated with porosity, indicating that the concrete with a water–cement ratio of 0.44 has the highest porosity, followed by the specimen with a 0.49 ratio, and the specimen with a 0.54 ratio has the lowest porosity. This is consistent with the law of energy evolution during the compression of concrete.

### 3.4. Damage Characteristics in the Compaction Stage

#### 3.4.1. Damage Characteristics Defined Based on Dissipated Energy

The variable that reflects the internal damage of concrete is termed the damage variable, and the water–cement ratio significantly impacts the porosity of the concrete [38]. The initial pores in the concrete before compression are a crucial reason for the nonlinearity in the pore compaction stage. Examining the damage characteristics in the compaction stage can highlight the influence of the water–cement ratio on the alteration of initial porosity and cracks in the concrete. The magnitude of dissipated energy directly reflects the extent of damage to the concrete specimen, and the damage of concrete during uniaxial compression contributes to the growth of dissipated energy. Hence, the damage variable *D* in the compression process of concrete is defined using dissipated energy as follows:

(5) $$D=\frac{U_{d}}{U_{d\max}}$$

where *U*_d_ represents the dissipated energy produced during the compression process of concrete; *U*_d__max_ signifies the maximum dissipated energy reached at the peak of concrete compression.

**Figure 11:**
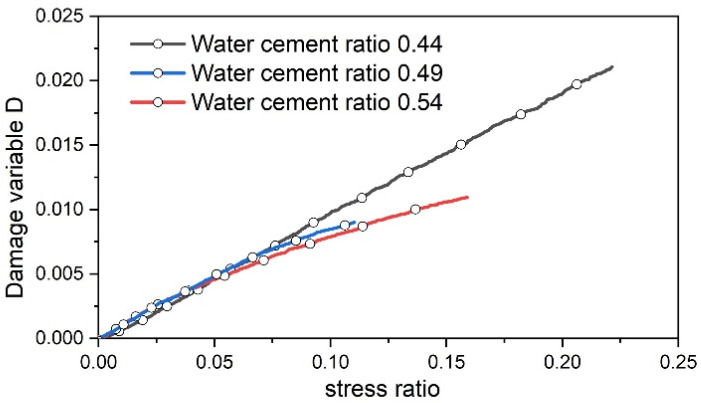
The dissipated energy defines the damage variable evolution diagram in the compaction stage.

Based on Equation (5), a diagram illustrating the evolution of the damage variable during the concrete pore compaction stage with varying water–cement ratios is depicted in Figure 11.

The primary mechanism underlying damage during the pore compaction stage stems from microplastic compaction. As depicted in Figure 11, the damage parameter of concrete specimens exhibits a modest increase, which is primarily due to the iterative compaction and closure of primary cracks within the concrete. As stress escalates, the damage parameter steadily rises, with concrete featuring a water–cement ratio of 0.44 demonstrating the highest growth rate, while that with a ratio of 0.54 displays the lowest growth rate. This phenomenon arises from the correlation between the growth rate of the damage parameter during the pore compaction stage and the plastic work exerted by external forces on the pores. Greater porosity corresponds to a heightened growth rate of the damage parameter. As the specimen approaches the elastic stage, the dissipative energy curve gradually levels off.

#### 3.4.2. Damage Characteristics Defined Based on Ultrasonic Wave Velocity

During the compression process of the concrete specimen, changes in ultrasonic wave velocity can reflect the progression of internal cracks within the specimen. Hence, the ultrasonic wave velocity can be utilized to define the concrete damage variable [39]:

(6) $$D=1-{\left(\frac{V_{p}}{V_{\mathrm{pf}}}\right)}^{2}$$

where *V*_p_ is the ultrasonic wave velocity of the concrete with microcracks; *V*_pf_ is the ultrasonic wave velocity of the concrete when it is not under pressure.

**Figure 12:**
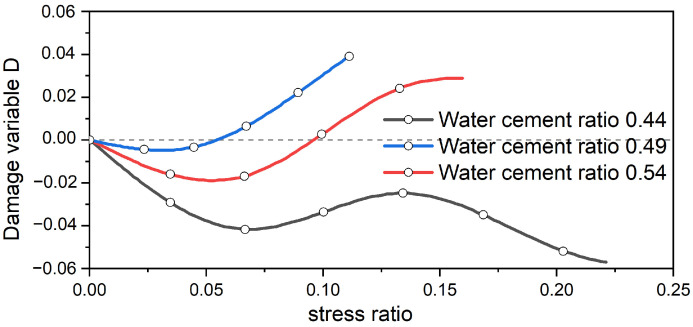
Wave velocity defines the evolution of damage variables in the compaction stage.

The damage variable graph for the pore compaction stage of concrete with different water–cement ratios is obtained using Equation (6), as shown in Figure 12.

The damage variable defined by ultrasound during the compaction stage is shown in Figure 12. It can be observed from Figure 12 that the damage variable curve takes on a wavelike shape. This is because ultrasound is a point measurement method, and the data obtained are point data. Additionally, concrete damage is an irreversible process, and ultrasound is affected by changes in the pores during the early stage of the concrete specimen’s compression. Therefore, the damage variable based on ultrasound velocity cannot accurately represent the changes in the concrete specimen during the compaction stage.

### 3.5. Analysis of Damage Characteristics

#### 3.5.1. Comparison of Damage Characteristics in the Compaction Stage

**Figure 13:**
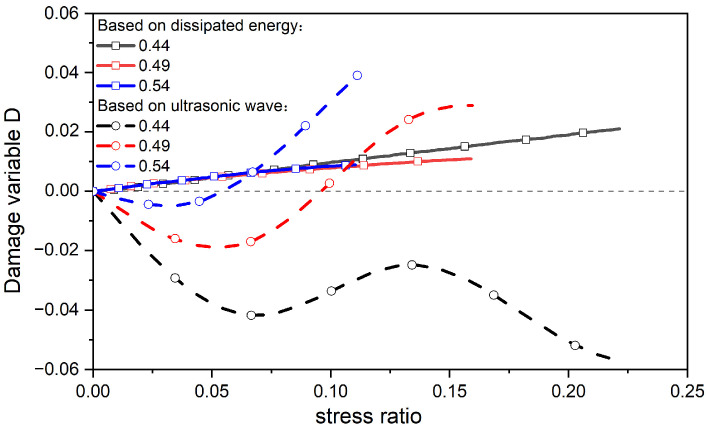
Comparison of damage characteristics in the compaction stage.

By integrating the damage variables defined based on dissipated energy and ultrasonic wave velocity, a comparison chart of the damage characteristics of concrete with diverse water–cement ratios during the compaction stage can be sketched, as presented in Figure 13.

From Figure 13, it can be observed that both the damage variable curve determined from dissipated energy and the one based on the alteration of ultrasonic wave velocity reflect the impact of the water–cement ratio on initial porosity. The porosity of the concrete specimen with a higher water–cement ratio is smaller. However, there are clear disparities between the damage variable curve defined by dissipated energy and the one defined by the change in ultrasonic wave velocity. The damage variable based on ultrasound is influenced by variations in porosity and assumes a wavelike form, failing to accurately represent the damage changes in the concrete specimen. Additionally, ultrasound measurement is a point-measuring approach, resulting in point data that are not precise enough. In contrast, the damage variable defined via dissipated energy is derived from the stress–strain curve, which involves continuous data. This method provides more accurate results, allowing a clear perception of the damage variable’s changing trend as stress increases, and showing the impact of different water–cement ratios on the concrete specimen. To sum up, during the compaction stage of concrete, the damage variable defined through dissipated energy based on the stress–strain curve is more effective.

#### 3.5.2. Whole Process Damage Variables Defined Based on Ultrasound

**Figure 14:**
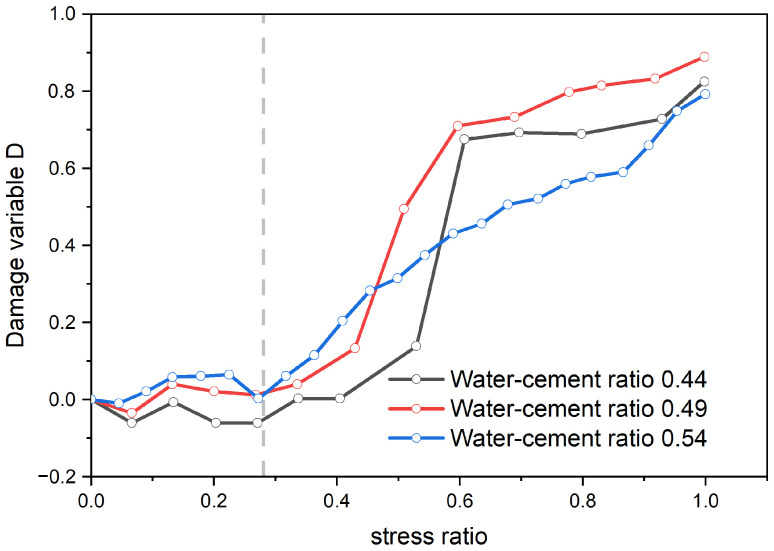
The full curve graph of the damage change evolution defined by wave velocity.

Since the damage variable defined by ultrasound cannot accurately reflect the damage characteristics during the concrete pore compaction stage, a complete curve diagram of the damage characteristic variable defined by ultrasound is sketched for discussion, as presented in Figure 14.

From Figure 14, it can be discerned that the overall alteration tendency of concrete specimens with diverse water–cement ratios is identical, and the damage variable curve generally increases with the increase in stress. There is a threshold for the damage variable of the concrete specimen. When the stress ratio is below this threshold, the damage variable assumes a wavelike form with no distinct change. When the stress ratio exceeds the threshold, the damage variable escalates rapidly. During the rapid increase in the damage variable, the ascending rate is greatest for the concrete with a water–cement ratio of 0.44, while it is smallest for the concrete with a ratio of 0.54. This indicates that the microcrack development rate in concrete specimens with a higher water–cement ratio is relatively sluggish, whereas specimens with a lower water–cement ratio develop a large number of microcracks in a short period. In conclusion, the damage variable defined by ultrasound can reflect the damage characteristics of the concrete specimen as a whole, which is consistent with the results obtained in the literature [39].

## 4. Discussion

This paper analyzes the influence of different water–cement ratios on concrete from two aspects: energy evolution and changes in ultrasonic wave velocity. The study found that increasing the water–cement ratio reduces the initial porosity of concrete, weakening its mechanical properties and diminishing the characteristics of the pore compaction stage. This is mainly because an excessively high water–cement ratio and sufficient hydration reaction reduce the initial porosity and mechanical properties of concrete, which is consistent with other researchers’ conclusions.

In the study of the damage characteristics of the pore compaction stage, the damage characteristic defined based on dissipated energy is more effective than that defined based on ultrasonic wave velocity. This is because energy evolution runs through the entire deformation and failure process of concrete, changes with porosity, and consists of continuous linear data, effectively reflecting damage changes in the pore compaction stage of concrete. Ultrasound, on the other hand, is affected by changes in the pore medium inside the concrete, causing wave velocity changes to appear wavy. Since concrete damage is an irreversible process, ultrasound cannot accurately reflect the damage characteristics of the pore compaction stage but is more suitable for reflecting the overall mechanical properties of concrete.

The influence of the water–cement ratio on concrete is mainly reflected in the pore compaction stage but is often overlooked. Therefore, this paper focuses on studying the damage characteristics of the pore compaction stage of concrete with different water–cement ratios, providing a reference for the detection and research of this stage.

This study conducted uniaxial compression tests on three common water–cement ratio concretes used in engineering, but the resulting theory has certain limitations. Further research and analysis on a broader range of water–cement ratio concretes are necessary. The definition of porosity is mainly obtained through energy evolution analysis and the comparison and verification of ultrasonic wave velocity to determine the size change relationship, but there is no precise porosity measurement. Conducting porosity measurement tests would aid in more accurate research. A comparative verification analysis of the damage characteristics of concrete during the uniaxial compression process was conducted based on the aspects of energy evolution and ultrasonic wave velocity. Defining the damage characteristics of concrete from multiple perspectives is crucial for ensuring the safety and durability of concrete.

## 5. Conclusions

This study conducted uniaxial compression tests on C30-grade concrete with water–cement ratios of 0.44, 0.49, and 0.54. The porosity inside the specimens was assessed through the comparison of energy evolution and ultrasonic signals. The ultrasonic detection results and the energy evolution law were used for mutual verification. The energy evolution and the change in ultrasonic wave velocity during the pore compaction stage of concrete with different water–cement ratios were thoroughly analyzed, and the characteristics of the damage variables defined from the perspectives of dissipated energy and ultrasonic wave velocity were compared. This study provides a reference for damage detection in the pore compaction stage of concrete with different water–cement ratios in practical engineering applications. The results are as follows:

1. The characteristics of the pore compaction stage for concrete specimens with a high water–cement ratio are not pronounced. As the water–cement ratio increases, the fluidity of the cement paste increases, forming a cementitious layer that blocks some pores, reducing the initial porosity of the concrete specimens and resulting in decreased mechanical properties.
2. In the pore compaction stage, the damage variable defined based on dissipated energy exhibits linear growth in the range of 0–0.025. The damage variable reflects the porosity of specimens with different water–cement ratios, and its growth trend reflects changes in porosity. Therefore, the damage variable defined based on dissipated energy can effectively reflect the damage characteristics of concrete specimens during the pore compaction stage.
3. In the pore compaction stage, due to changes in the medium inside the pores, the ultrasonic wave undergoes diffraction, resulting in the fluctuation of the damage variable curve in the range of −0.06 to 0.04. Moreover, the damage to concrete is an irreversible process. Therefore, the damage variable defined based on ultrasound cannot accurately reflect the damage characteristics of the pore compaction stage.
4. In the pre-peak stress stage, the damage variable defined based on ultrasound is in the range of −0.1 to 0.9, with an obvious threshold (the stress ratio is about 0.3). When the stress ratio is less than the threshold, the damage variable curve is wavy and has no obvious pattern; when the stress ratio is greater than the threshold, the damage variable increases rapidly, and its growth trend can reflect changes in the internal cracks within the specimens. Therefore, the damage variable defined based on ultrasound can reflect the overall mechanical properties of concrete.

## Figures and Tables

**Table 1:** Basic performance table of cement.

Setting Time (Min)		Flexural Strength (Mpa)		Compressive Strength (Mpa)	
Initial Setting Specimen	Final Setting Time	3 Day	28 Day	3 Day	28 Day
>150	<250	4.5	7.5	22	46

**Table 2:** The basic performance of crushed stone.

Water Absorption Rate (%)	Apparent Density (kg/m^−3^)	Bulk Density (kg/m^−3^)	Void Fraction (%)
1.6	2700	14,600	38.9

**Table 3:** Concrete test mix ratio.

Water–Cement Ratio	Cement (kg/m^3^)	Water (kg/m^3^)	Gravel (kg/m^3^)	Sand (kg/m^3^)
0.44	450	200	1200	650
0.49	400	195	1200	650
0.54	350	190	1200	650

## Data Availability

The original contributions presented in the study are included in the article; further inquiries can be directed to the corresponding author/s.
